# Effect of vitamin E on oxidative stress level in blood, synovial fluid, and synovial tissue in severe knee osteoarthritis: a randomized controlled study

**DOI:** 10.1186/s12891-017-1637-7

**Published:** 2017-06-29

**Authors:** Saran Tantavisut, Aree Tanavalee, Sittisak Honsawek, Tanyawan Suantawee, Srihatach Ngarmukos, Sirichai Adisakwatana, John J. Callaghan

**Affiliations:** 1 Department of Orthopaedics, Faculty of Medicine, Chulalongkorn University, 1873 Rama 4 road, Pathumwan, Bangkok, 10330 Thailand; 2 Department of Biochemistry, Faculty of Medicine, Chulalongkorn University, Bangkok, Thailand; 3 Faculty of Allied Health Sciences, Chulalongkorn University, Bangkok, Thailand; 4 Department of Orthopaedics, Faculty of Medicine, University of Iowa Hospitals and Clinics, Iowa City, IA USA

**Keywords:** Knee osteoarthritis, Oxidative stress, Antioxidant, Anti-inflammation, Vitamin E

## Abstract

**Background:**

This study was performed to evaluate the antioxidative and anti-inflammatory effects of vitamin E on oxidative stress in the plasma, synovial fluid, and synovial tissue of patients with knee osteoarthritis.

**Methods:**

Seventy-two patients with late-stage knee osteoarthritis scheduled for total knee arthroplasty were randomized to take oral placebo (Group A) or 400 IU of vitamin E (Group B) once a day for 2 months before undergoing surgery. The blood levels of endpoints indicating oxidative stress or antioxidant capacity, Knee Society Score (KSS), Western Ontario and McMaster Universities Osteoarthritis Index score (WOMAC), and adverse effects were compared before and after the intervention between the two groups. At surgery, these redox endpoints and histological findings were compared between the synovial fluid and synovial tissue.

**Results:**

In blood samples, the pre-intervention of oxidative stress and antioxidative capacity were not different between Group A and Group B. In post-intervention blood samples, the Malondialdehyde (Group A 1.34 ± 0.10, Group B 1.00 ± 0.09, *p* < 0.02), Alpha tocopherol (Group A 15.92 ± 1.08, Group B 24.65 ± 1.47, *p* < 0.01) and Trolox equivalent antioxidant capacity (Group A 4.22 ± 0.10, Group B 5.04 ± 0.10, 0 < 0.01) were significantly different between Group A and Group B. In synovial fluid samples, the Malondialdehyde (Group A 1.42 ± 0.12, Group B 1.06 ± 1.08, p 0.01), Alphatocopherol (Group A 4.51, Group B 7.03, *p* < 0.01), Trolox equivalent antioxidant capacity (Group A, 1.89 ± 0.06, Group B 2.19 ± 0.10) were significantly different between Group A and Group B. The pre-intervention WOMAC score and KSS score were not different between Group A and Group B. The post-intervention WOMAC score was significantly improved in all categories in Group B (Pain: Group A 27.26 ± 0.89, Group B 19.19 ± 1.43, *p* < 0.01; Stiffness: Group A 8.23 ± 0.79, Group B 5.45 ± 0.73, p 0.01; Function: Group A 94.77 ± 4.22, Group B 72.74 ± 6.55, *p* < 0.01). The post-intervention KSS score was significantly improved in all categories in Group B (Clinical: Group A 25.31 ± 14.33, Group B 33.52 ± 16.96, *p* < 0.01; Functional: Group A 41.43 ± 16.11, Group B 51.61 ± 19.60, p 0.02). Significantly fewer synovial tissue cells were stained with nitrotyrosine and hematoxylin–eosin in Group B than in Group A. There were no differences in adverse effects or surgical complications between the groups.

**Conclusion:**

Vitamin E is an effective antioxidant that can improve clinical symptoms and reduce oxidative stress conditions in patients with late-stage knee osteoarthritis.

**Trial registration:**

This research project had been approved for registration at Thai Clinical Trials Registry (TCTR) since 2016–08-28 11:26:32 (Retrospective registered). The TCTR identification number is TCTR20160828001.

## Background

Although osteoarthritis (OA) of the knee is a major cause of chronic disability in the older population, full comprehension of the pathogenesis of this disease has proven elusive [1, 2]. Recent evidence demonstrates that oxidative stress, the condition wherein oxidant levels exceed those of antioxidative agents, is one of the inducing factors of OA [3–9]. Reactive oxygen species, including oxidants that are produced under physiological conditions in the human body and removed by cellular antioxidants, lead to structural and functional damage of cartilage cells. Several laboratory investigations of the relationship between oxidative stress and OA have been undertaken. Increased nitrite, a stable decomposition biochemical marker of the presence of nitric oxide, has been reported in the plasma and synovial fluid of patients with OA [5]. In addition, several oxidative damage end-products following intracellular molecular oxidative stress have been identified, such as malondialdehyde (MDA) and nitrotyrosine. Elevated levels of MDA have been reported in the plasma of patients with OA [6]. Notably, patients with knee OA have higher levels of plasma oxidative stress parameters but lower levels of plasma antioxidant parameters than those of healthy controls [8]. The combined antioxidant effects of these antioxidative agents have been described as the total antioxidant capacity. Assays of the ferric reducing antioxidant power (FRAP) and Trolox equivalent antioxidant capacity (TEAC) have been utilized in this manner to measure the total antioxidant status [8].

Vitamin E, a dietary antioxidant capable of augmenting the total cellular antioxidant capacity, reportedly has a positive effect on the symptomatic treatment of arthritis [10–15]. However, there is very little evidence from high quality trials that vitamin E modifies oxidative markers and symptoms in people with knee osteoarthritis [16, 17]. This is the first randomized controlled trial that focus on the effects of vitamin E in end stage knee OA and completely evaluates clinical symptoms, biochemistry and histology results. We hypothesized that a sustained duration of vitamin E administration will decrease the oxidative stress, inflammatory process and improve symptoms in patients with end stage knee OA. Therefore, the purpose of this study was to evaluate the antioxidative and anti-inflammatory effects of vitamin E on oxidative stress in the plasma, synovial fluid, and synovial tissue of patients with knee OA.

## Methods

### Study design

The level of evidence in this study is level I. This study was approved by the Institutional Review Board on Human Research of the Faculty of Medicine, Chulalongkorn University (reference number 412/52) and was conducted in compliance with the guidelines of the Declaration of Helsinki. Written informed consent was obtained from the patients prior to their participation in the study. This research project had been approved for registration at Thai Clinical Trials Registry (TCTR) since 2016–08-28 11:26:32 (Retrospective registered). The TCTR identification number is TCTR20160828001. This single-center, randomized, double-blinded, placebo-controlled trial was performed at our institution from 1 July 2011 through 31 July 2012. An independent safety monitoring board evaluated the patients’ safety and conducted semiannual meetings during the trial.

### Participants

Eligible patients were ≥18 years of age, clinically diagnosed with knee OA according to the criteria of the American College of Rheumatology [18], radiographically diagnosed with severe knee OA (grade 3–4) as defined by the Kellgren–Lawrence classification [19], and scheduled for total knee arthroplasty (TKA) within 2 months (60 ± 2 days) after enrollment. Patients were excluded if they had an established diagnosis of a bleeding disorder; currently used vitamin E-containing drugs or dietary supplements, anticoagulants, or antiplatelet therapy; or had a vitamin E allergy. The use of nonsteroidal anti-inflammatory drugs was not allowed during the study period. The participants were allowed to use only acetaminophen 500 mg 1–2 tablets (depending on participant’s weight) every 6 h as needed for pain.

### Randomization and blinding

Using sealed envelopes, participants were randomly assigned by independent outpatient unit nurses to one of two groups in a 1:1 ratio: those who received placebo per oral once daily for 2 months (Group A) and those who received 400 IU of vitamin E per oral once daily for 2 months (Group B). The placebo and its container have identical color, form and size as the vitamin E. The surgeons and care team, all investigators, participants, staff collecting data, statistician and staff performing assays were blinded from the randomization.

### Outcome measurements

The primary outcome measurements are biochemistry and histology results. The secondary outcome measurements are clinical results. In both groups of participants, the modified Thai Western Ontario and McMaster Universities Osteoarthritis Index Score (WOMAC) [20], Knee Society Score (KSS), and blood samples were collected before participants took the first dose of the assigned treatment (baseline) and at 2 months of drug administration, just prior to the surgery (post-intervention). Adverse events and surgical complications were recorded from the first dose of the assigned treatment until 2 weeks postoperatively. All patients underwent TKA within 2 days after completion of the 2-month course of vitamin E or placebo. Our calculation showed that 33 participants in each treatment group would provide the study with ≥80% power to detect a mean between-group difference (calculated with a standard deviation of 1.6 from our pilot study and a two-sided type I error rate of 0.05). The estimated mean between group differences that could be detected is 1.21. Considering a drop-out rate of 10%, the total sample size required was 72 (36 in each arm).

### Specimen collection and laboratory methods

A 5-ml venous blood sample was collected from the cubital vein of each patient at baseline and postintervention. All samples were centrifuged at 4000 rpm for 10 min and stored immediately at −80 °C until analysis. The synovial fluid was aspirated prior to opening of the knee. The synovial tissue was collected from the most inflamed and redness area of the anterior femoral fat pad during TKA. The synovial fluid was then instantly centrifuged to remove cells and joint debris. Similarly, both the synovial fluid and tissue specimens were stored at −80 °C until sample collection from the last patient had been completed. Multiple investigations of oxidative stress, vitamin E, and antioxidant capacity were performed as described below.

### Determination of nitrite concentrations [21]

The nitrite concentrations in plasma and synovial fluid were measured with the Griess Reagent System (Promega, Madison, WI, USA) using sulfanilamide and N-1-napthylenediamine dihydrochloride under acidic (phosphoric acid) conditions. Formation of the azo compound was determined via its absorbance at 540 nm by spectrophotometry.

### Determination of MDA concentration

The MDA concentrations in plasma and synovial fluid were determined by the reaction between MDA and 2-thiobarbituric acid (Sigma-Aldrich, St. Louis, MO, USA) at 95 °C [22]. MDA and 2-thiobarbituric acid react with each other to produce a pink chromogen with absorbance at 532 nm by spectrophotometry. Malondialdehyde tetrabutylammonium salt (Sigma-Aldrich) was used as standard MDA.

### Determination of vitamin E concentration by reverse-phase high-performance liquid chromatography

Plasma or synovial fluid samples were extracted with ethanol (Merck, Darmstadt, Germany) and hexane (Merck). Alpha-tocopheryl acetate (Sigma-Aldrich) was used as internal standard. We used alpha tocopherol (258,024 Sigma) as an external standard to make a standard curve. Then we calculated vitamin E concentrations from that standard curve. After drying the hexane layer under nitrogen gas and resuspending it in methanol (Merck), the extract was injected into the high-performance liquid chromatography (HPLC) system. The stationary phase was established using a C18 column (Inertsil ODS-3; GL Sciences, Tokyo, Japan). The mobile phase was a mixture of isocratic methanol/water (98/2, *v*/v) at a flow rate of 1.5 ml/min. The HPLC peaks were detected with an ultraviolet detector at 292 nm [23].

### Determination of total antioxidant capacity by FRAP

The total antioxidant capacity was measured by FRAP assay as described by Benzie and Strain [24]. This assay depends upon the reduction of ferric tripyridyltriazine complex (Sigma-Aldrich) to ferrous tripyridyltriazine form at low pH. After this reduction, the absorbance was determined at 593 nm. Ferrous sulfate (Sigma-Aldrich) was used as the standard.

### Determination of total antioxidant capacity by TEAC assay [25, 26]

The TEAC assay is based on the samples’ scavenging of the 2,2′-azinobis-(3-ethylbenzothiazoline-6-sulfonic acid) (Sigma-Aldrich) radical anion, which is converted into a colorless product. The decrease in absorption at 734 nm after 6 min of the addition of a test compound was used to calculate the TEAC values. Different concentrations of hydroxy-2,5,7,8-tetramethylchromane-2-carboxylic acid (Trolox) (Sigma-Aldrich) were used to create a calibration curve.

### Determination of tissue nitrotyrosine concentration [27]

Synovial tissues were fixed in neutral buffered formalin, embedded in paraffin, and cut into 4-μm sections. Nitrotyrosine immunohistochemistry was performed using rabbit polyclonal anti-nitrotyrosine antibody (Upstate Biotechnology, Lake Placid, USA) as the primary antibody followed by anti-mouse/anti-rabbit immunoglobulins (Dako Envision System, Dako, Denmark) conjugated to peroxidase enzyme. For secondary antibody evaluation, the immunolabeling results of both groups were compared by direct visual inspection.

### Determination of inflammatory infiltration of synovial tissue

Synovial tissues were fixed and embedded in paraffin then cut into 4-μm sections as above. Slides were stained with hematoxylin and eosin (H&E), and inflammatory cells were counted using a light microscope at 40× magnification (randomly selected 10 fields/specimen).

### Statistical analysis

The baseline demographic data and patient characteristics were compared between the treatment groups. Categorical data were analyzed with the chi-square test or Fisher’s exact test as appropriate. Continuous variables were analyzed using independent sample t-test or the Wilcoxon rank sum test. All *p* values were two-sided. A *p* value of ≤0.05 was considered to indicate statistical significance. All computations were carried out using GraphPad Prism 5.0.1 (GraphPad Software Inc., La Jolla, CA, USA).

## Results

**Fig. 1:**
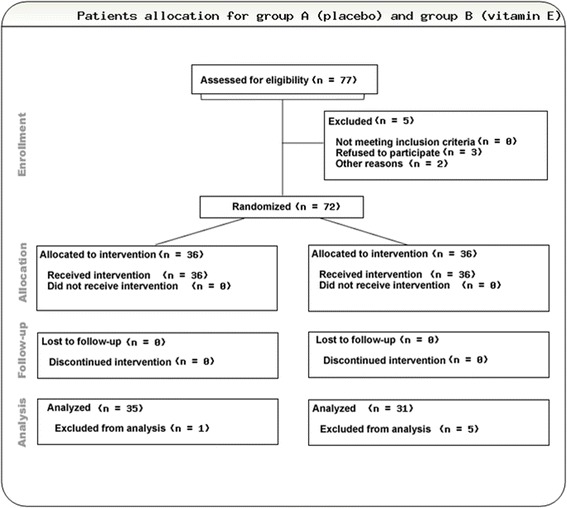
The CONSORT flow diagram of the studied group

The CONSORT flow diagram of the studied cohort is shown in Fig. 1. At the time of analysis, six patients were excluded (one in Group A and five in Group B) because the operative date was delayed for more than 2 days from the scheduled date. The delay in surgery were because of personal reasons and not related to the trial or knee symptoms. Thus, there were 35 patients in Group A (placebo) and 31 patients in Group B (vitamin E). All participants were fully adherent to their interventions.

### Baseline characteristics

The baseline comparative demographic data between the patients with knee OA in Group A (placebo) and Group B (vitamin E) are shown in Table 1. There were no significant differences in age, sex, or body mass index between the two groups. Sex was not a potential confounder in this study based on the absence of any differences in treatment response between males and females. Similarly, the baseline preoperative WOMAC score (Table 2) and KSS score (Table 3) were not significantly different between the two groups.

**Table 1:** Baseline demographic data of patients

	Group A	Group B	*p* value
Number of patients	35	31	–
Age, years	69.20 ± 1.30	69.50 ± 1.30	0.87
Sex			
Male/female	9/26	2/29	–
Weight, kg	63.90 ± 1.60	60.10 ± 2.00	0.13
Height, cm	155.10 ± 1.30	154.80 ± 1.20	0.90
Body mass index, kg/m^2^	26.60 ± 0.60	25.00 ± 0.40	0.09
Kellgren–Lawrence grading			
Grade 3	10	9	–
Grade 4	25	22	–

Data are presented as number of patients or mean ± standard deviation

**Table 2:** Western Ontario and McMaster Universities Osteoarthritis Index Score

	Group A (placebo)	Group B (vitamin E)	*p* value
Pain (50 points)			
Pre-intervention	25.20 ± 1.19	23.77 ± 1.69	0.49
Post-intervention	27.26 ± 0.89	19.19 ± 1.43	<0.01^a^
∆ (post −pre)	2.06 ± 0.88	−4.581 ± 1.11	<0.01^a^
Stiffness (20 points)			
Pre-intervention	7.80 ± 0.87	8.94 ± 1.02	0.39
Post-intervention	8.23 ± 0.79	5.45 ± 0.73	0.01^a^
∆ (post − pre)	0.43 ± 0.73	−3.48 ± 0.77	<0.01^a^
Function (170 points)			
Pre-intervention	90.06 ± 4.72	90.55 ± 7.12	0.95
Post-intervention	94.77 ± 4.22	72.74 ± 6.55	<0.01^a^
∆ (post − pre)	4.71 ± 3.81	−17.81 ± 4.23	<0.01^a^

Data are presented as mean ± standard deviation

*P* value represent the test between groups

^a^Statistically significant

**Table 3:** Clinical and functional Knee Society Scores

	Group A (placebo)	Group B (vitamin E)	*p* value
Clinical			
Pre-intervention	23.20 ± 14.52	22.48 ± 16.66	0.85
Post-intervention	25.31 ± 14.33	33.52 ± 16.96	<0.01^a^
∆ (post − pre)	2.11 ± 7.26	11.03 ± 8.561	<0.01^a^
Functional			
Pre-intervention	41.14 ± 16.05	41.94 ± 18.20	0.85
Post-intervention	41.43 ± 16.11	51.61 ± 19.60	0.02^a^
∆ (post − pre)	0.29 ± 1.312	9.68 ± 1.99	<0.01^a^

Data are presented as mean ± standard deviation

*P* value represent the test between groups

^a^Statistically significant

### Oxidative stress, vitamin E, and antioxidant capacity in plasma and synovial fluid

**Fig. 2:**
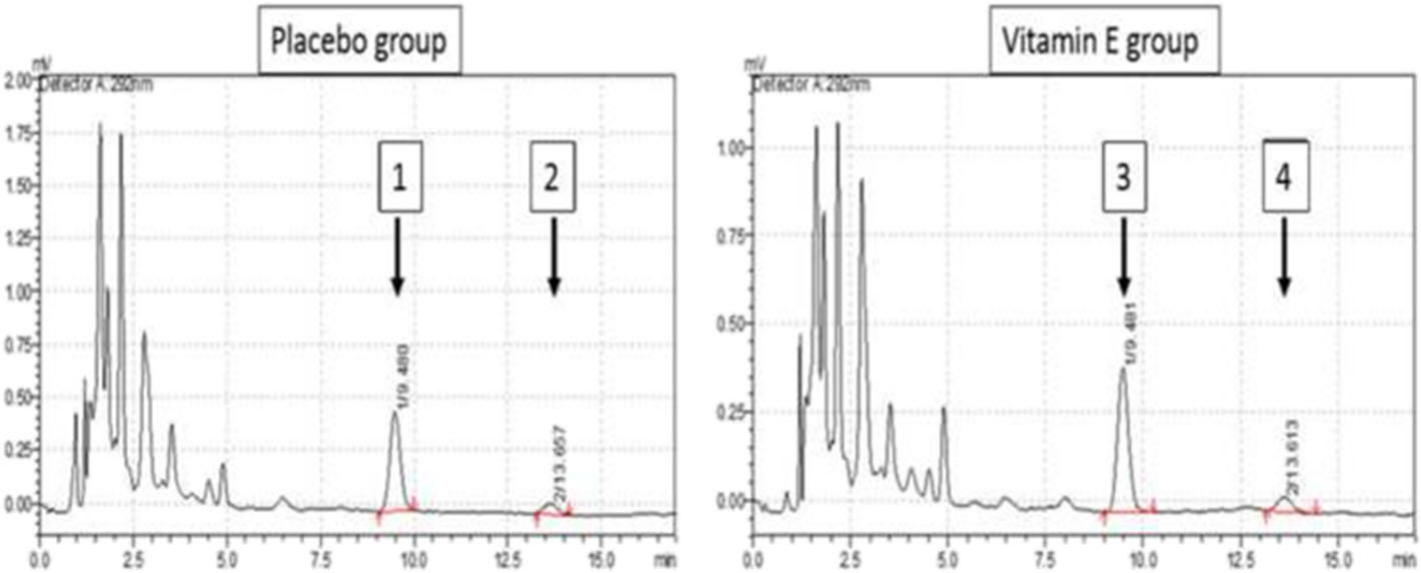
The HPLC chromatogram from serum samples demonstrates “peak 2” and “peak 4” which represent alpha tocopheryl acetate (internal standard) in group A and group B respectively; “Peak 1” demonstrates alpha tocopherol in group A; “Peak 3” demonstrates alpha tocopherol in group B

The HPLC chromatogram is shown in Fig. 2. Changes in the oxidative stress level in plasma between before and after the intervention with each tested agent are shown in Table 4. The post-intervention nitrite concentration in Group B was lower than that in Group A, but the difference was not significant (*p* = 0.19); however, the post-intervention MDA level in Group B was significantly lower than that in Group A (*p* = 0.02). The post-intervention levels of vitamin E (alpha-tocopherol) and TEAC in Group A increased to a significantly lesser degree than those in Group B (*p* < 0.01 and <0.01, respectively). In contrast, the post-intervention changes in the antioxidant capacity using the FRAP assay were not different between the two groups. The synovial fluid analysis results were similar to the serum analysis results, as shown in Table 5.

**Table 4:** Oxidative stress and antioxidative capacity measurements from blood samples

	Group A (placebo)	Group B (vitamin E)	*p* value
Oxidative agents and products			
- Nitrite (μM)			
Pre-intervention	5.55 ± 0.57	5.60 ± 1.09	0.96
Post-intervention	5.63 ± 0.58	3.89 ± 1.25	0.19
∆ (post − pre)	0.08 ± 0.03	−1.70 ± 1.2	0.12
- Malondialdehyde (μM)			
Pre-intervention	1.32 ± 0.11	1.43 ± 0.07	0.43
Post-intervention	1.34 ± 0.10	1.00 ± 0.09	0.02^a^
∆ (post − pre)	0.01 ± 0.02	−0.79 ± 0.39	0.03^a^
Antioxidative agents			
- Alpha-tocophorol (μg/ml)			
Pre-intervention	15.50 ± 0.98	16.24 ± 0.99	0.59
Post-intervention	15.92 ± 1.08	24.65 ± 1.47	<0.01^a^
∆ (post − pre)	0.43 ± 0.33	8.41 ± 1.18	<0.01^a^
- FRAP (mM)			
Pre-intervention	923.30 ± 29.46	905.20 ± 35.16	0.69
Post-intervention	951.30 ± 29.03	1020 ± 40.22	0.17
∆ (post − pre)	28.01 ± 9.11	114.4 ± 35.19	0.01^a^
- TEAC (mM)			
Pre-intervention	4.26 ± 0.10	4.56 ± 0.14	0.08
Post-intervention	4.22 ± 0.10	5.04 ± 0.10	<0.01^a^
∆ (post − pre)	−0.04 ± 0.05	0.89 ± 0.45	0.03^a^

Data are presented as mean ± standard deviation

*P* value represent the test between groups

*FRAP* ferric reducing antioxidant power, *TEAC* Trolox equivalent antioxidant capacity

^a^Statistically significant

**Table 5:** Oxidative stress and antioxidative capacity measurements from synovial fluid

	Group A (placebo)	Group B (vitamin E)	*P* value
Oxidative agents and products			
- Nitrite (μM) - Malondialdehyde (μM)	5.94 ± 1.09 1.42 ± 0.12	5.24 ± 0.91 1.06 ± 1.08	0.63 0.01^a^
Antioxidative agents			
- Alpha-tocopherol (μg/ml) - FRAP (mM) - TEAC (mM)	4.51 ± 0.31 999.10 ± 39.51 1.89 ± 0.06	7.03 ± 0.86 1103 ± 44.77 2.19 ± 0.1	<0.01^a^ 0.09 0.01^a^

Data are presented as mean ± standard deviation

*P* value represent the test between groups

*FRAP* ferric reducing antioxidant power, *TEAC* Trolox equivalent antioxidant capacity

^a^Statistically significant

Correlation analysis of the differences between postoperative and preoperative (Delta, Δ) of oxidative stresses, antioxidative capacity and clinical outcomes in group B were performed. The results are shown in Table 6. The significant inverse correlation of Δ TEAC and Δ WOMAC score was found (*p* = 0.03). In addition, the significant inverse correlation of Δ TEAC and Δ MDA was also demonstrated.

**Table 6:** Correlation analysis of the differences between preintervention and postintervention (Delta, Δ) of oxidative stresses, antioxidative capacity and clinical outcomes in the serum samples of group B

		Δ WOMAC	Δ KSS	Δ MDA	Δ TEAC	Δ Vit E	Δ Nitrite
Δ WOMAC	C*	1	−0.08	0.31	−0.40	−0.09	−0.02
	P**	-	0.69	0.09	0.03*	0.62	0.91
Δ KSS	C*	−0.08	1	−0.16	0.21	−0.10	−0.17
	P**	0.69	-	0.40	0.26	0.96	0.35
Δ MDA	C*	0.31	−0.16	1	−0.38	−0.22	−0.03
	P**	0.09	0.40	-	0.04*	0.24	0.89
Δ TEAC	C*	−0.40	0.21	−0.38	1	0.10	0.25
	P**	0.03	0.26	0.04	-	0.58	0.18
Δ Vit. E	C*	−0.09	−0.10	−2.2	0.10	1	0.09
	P**	0.62	0.96	0.24	0.58	-	0.61
Δ Nitrite	C*	−0.02	−0.17	−0.03	0.25	0.09	1
	P**	0.91	0.35	0.89	0.18	0.61	-

C* = Pearson correlation

P** = *p* value

### Histological evaluation of synovial tissue

**Fig. 3:**
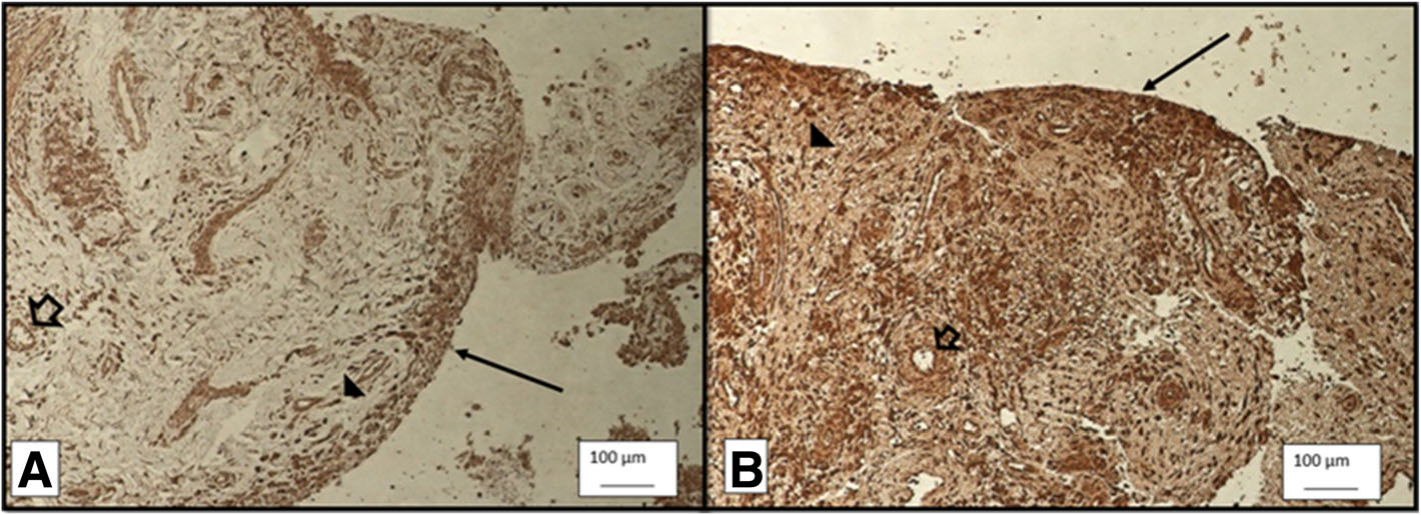
Oxidative stress marker Nitrotyrosine stain under 10X light microscope in group A (**a**) and group B (**b**). The nitrotyrosine staining in Group A (3A) was substantially more prominent than that in Group B (3B). In both groups, the staining was prominent at synovial lining cells and subsynovium area (*arrow*), synoviocytes (*arrow head*) and capillary endothelial cells (*hollow arrow*)

**Fig. 4:**
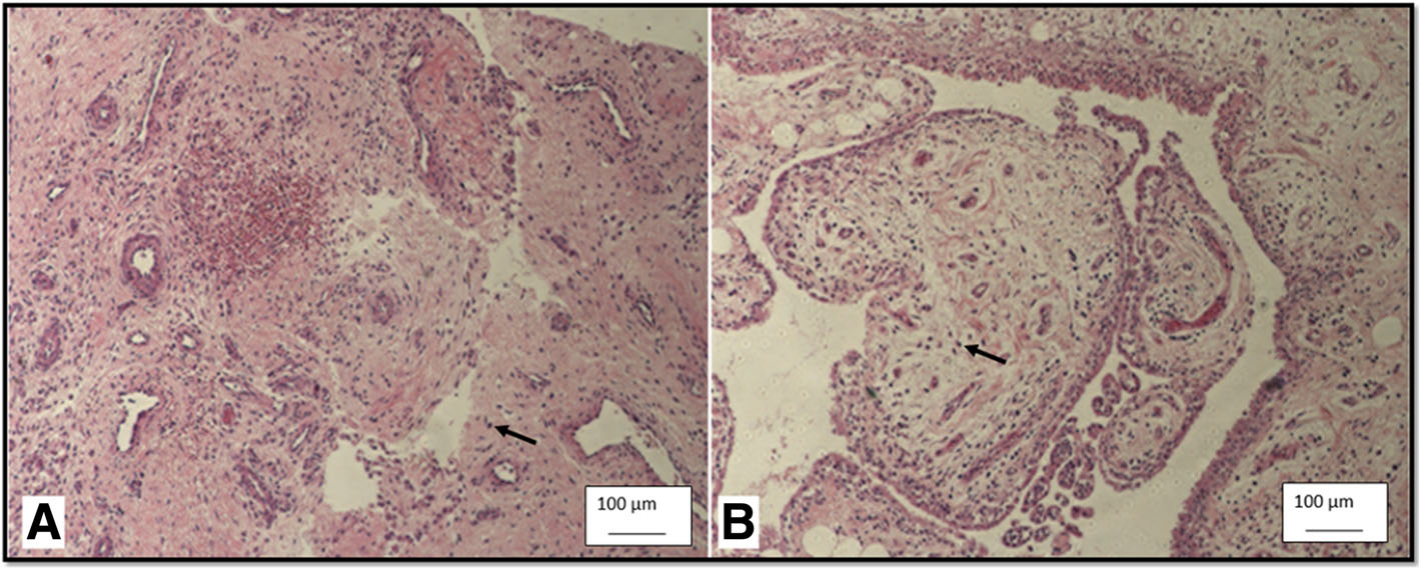
The Hematoxylin and Eosin stain under 10X light microscope demonstrates inflammation cells which were stained in *blue* (*arrow*). The mean inflammation cells were 15.33 ± 1.19 cells/high power field in group A (**a**) and 9.47 ± 0.54 cells/high power field in group B (**b**) with statistical significance (*p < 0.01*)

Immunohistochemical analyses of synovial tissue revealed that the nitrotyrosine staining in Group A was substantially more prominent than that in Group B (Fig. 3). In both groups, the staining was prominent at synovial lining cells, subsynovium area, synoviocytes and capillary endothelial cells. In addition, the H&E-stained slides showed significantly higher numbers of inflammatory cells in Group A than in Group B (Fig. 4).

### Clinical outcomes

The post-intervention WOMAC score was significantly improved in all categories in Group B (Pain: Group A 27.26 ± 0.89, Group B 19.19 ± 1.43, *p* < 0.01; Stiffness: Group A 8.23 ± 0.79, Group B 5.45 ± 0.73, *p* = 0.01; Function: Group A 94.77 ± 4.22, Group B 72.74 ± 6.55, *p* < 0.01). The post-intervention KSS score was significantly improved in all categories in Group B (Clinical: Group A 25.31 ± 14.33, Group B 33.52 ± 16.96, *p* < 0.01; Functional: Group A 41.43 ± 16.11, Group B 51.61 ± 19.60, *p* = 0.02). There were no differences in adverse events or surgical complications between the two groups. The adverse events were diarrhea (2.9% in Group A, 6.5% in Group B), minor prolonged wound bleeding (2.8% in Group A, 3.2% in Group B), and nausea/vomiting (5.7% in Group A, 3.2% in Group B).

## Discussion

Oxidative agents such as nitrite [2, 3, 28] and MDA [29], antioxidants such as vitamin E [30–32], and the total antioxidant capacity [32] as indicated by TEAC and FRAP have been investigated in patients with OA. In the present study, participants in Group B demonstrated lower levels of all oxidant endpoints in both the plasma and synovial fluid than did participants in group A; the differences were both statistically significant (MDA in plasma and synovial fluid) and statistically insignificant (nitrite). The nitric oxide is produced by the synovium and cells such as endothelial cells, polymorphonuclear leucocytes and macrophages. These cells are increased in inflamed synovial tissue. This phenomenon may explain the prominent nitrotyrosine stained in synovium lining and subsynovium tissue. The inflamed synovial tissue with intense nitric oxide production may obviously reaction to vitamin E leading to markedly decrease nitrotyrosine stain in group B tissue. The synovial fluid contains less amount of nitric oxide comparing with synovial tissue.

With respect to the antioxidant and total antioxidant capacity, the levels of vitamin E (alpha-tocopherol), FRAP, and TEAC in Group B were higher than those in Group A in both the plasma and synovial fluid. However, statistical significance between the two groups was found in the levels of vitamin E (*p* = < 0.01 in serum and *p* = < 0.01 in synovial fluid) and TEAC (*p* < 0.01 in serum and synovial fluid). These findings are in agreement with several previous studies [13, 14].

The changes in oxidative stress, the antioxidant level, and the total antioxidant capacity in the serum samples were in agreement with the changes in clinical outcomes: patients in Group B showed significant improvement in all entities of the WOMAC scores (pain, stiffness, and functional parts), as well as in the clinical and functional KSS, while there were no changes in these clinical scoring systems in Group A. The correlation analysis in this study also support that the increasing of TEAC significantly correlate with improvement of the WOMAC score. Vasanthi et al. [13], concluded that vitamin E has an analgesic effect by suppression of nitric oxide and protein kinase C, which might desensitize the central pain pathway. The improvement of both clinical scores in this research possibly be related to the analgesic effect from the vitamin E itself, increasing of antioxidant and decreasing of oxidative level.

Furthermore, the histological study using H&E staining of synovial tissue in the current study found significantly lower inflammation in samples of Group B than those of Group A. This finding is in agreement with several previous studies reporting that vitamin E, as a potent antioxidant, has anti-inflammatory effects on knee tissues [13, 33, 34]. Thus, the lower inflammation in Group B might have improved patient pain and played an important role in improving the WOMAC score and KSS in the present study.

The significantly increased level of vitamin E (alpha-tocopherol) in the serum and synovial fluid samples in this study suggests that 400 IU of vitamin E daily for 2 months is well distributed into both the blood system and target site within the knee joint without any serious side effects or complications related to treatment. In the present study, vitamin E demonstrated the ability to decrease oxidative stress and increase the total antioxidant power in patients with knee OA. Thus, vitamin E may have the potential to act as a disease-modifying agent for OA.

The strengths of the present study include its randomized design allowing for the evaluation of clinical outcomes, laboratory findings, and histological findings of all patients simultaneously after the intervention. Thus, the combination of the evaluated parameters provides high reliability. However, the major weakness of the present study was the relatively short-term vitamin E administration (2 months) in patients with severe knee OA that had developed over a lifetime. Further studies involving patients with less severe OA with a focus on the application time and duration of vitamin E supplementation should be performed to determine whether vitamin E is a viable nonsurgical intervention for this debilitating disease.

## Conclusions

Vitamin E is an effective antioxidant that can safely improve clinical symptoms and reduce oxidative stress conditions in patients with late-stage knee osteoarthritis.

## Abbreviations

FRAP: Ferric reducing antioxidant power
H&E stained: Hematoxylin and Eosin stained
HPLC: High-performance liquid chromatography
KSS: Knee society score
MDA: Malondialdehyde
OA: Osteoarthritis
TCTR: Thai Clinical Trials Registry
TEAC: Trolox equivalent antioxidant capacity
TKA: Total knee arthroplasty
WOMAC: The Western Ontario and McMaster Universities arthritis score
